# Randomized, phase II trial of sequential hepatic arterial infusion chemotherapy and sorafenib versus sorafenib alone as initial therapy for advanced hepatocellular carcinoma: SCOOP-2 trial

**DOI:** 10.1186/s12885-019-6198-8

**Published:** 2019-10-15

**Authors:** Masaaki Kondo, Manabu Morimoto, Satoshi Kobayashi, Shinichi Ohkawa, Hisashi Hidaka, Takahide Nakazawa, Hiroshi Aikata, Takeshi Hatanaka, Daichi Takizawa, Kotaro Matsunaga, Chiaki Okuse, Michihiro Suzuki, Masataka Taguri, Takako Ishibashi, Kazushi Numata, Shin Maeda, Katsuaki Tanaka

**Affiliations:** 1 Gastroenterological Center, Yokohama City University Medical Center 4-57, Urafune-cho, Minami-ku, Yokohama, Kanagawa 232-0024 Japan; 2 Department of Gastroenterology, Yokohama City University Hospital; 3-9, Fukuura, Kanazawa-ku, Yokohama, Kanagawa 236-0004 Japan; 3 Hepatobiliary and Pancreatic Medical Oncology, Kanagawa Cancer Center Hospital; 1-1-2, Nakao, Asahi-ku, Yokohama, Kanagawa 241-0815 Japan; 4 Gastroenterology Division of Internal Medicine, Kitasato University Hospital; 1-15-1, Kitasato, Minami-ku, Sagamihara, Kanagawa 252-0329 Japan; 5 Department of Gastroenterology and Metabolism, Applied Life Sciences, Institute of Biomedical and Health Sciences, Hiroshima University; 1-2-3, Kasumi, Minami-ku, Hiroshima, Hiroshima 734-8551 Japan; 6 Department of Internal Medicine, Isesaki Municipal Hospital; 12-1, Tsunatorihonmachi, Isesaki, Gunma 372-0817 Japan; 7 Division of Gastroenterology and Hepatology, Department of Internal Medicine, St Marianna University School of Medicine; 2-16-1, Sugao, Miyamae-ku, Kawasaki, Kanagawa 216-8511 Japan; 8 Division of Gastroenterology and Hepatology, Kawasaki Municipal Tama Hospital; 1-30-37, Shukugawara, Tama-ku, Kawasaki, Kanagawa 214-8525 Japan; 9 Department of Data Science, Yokohama City University School of Data Science; 3-9, Fukuura, Kanazawa-ku, Yokohama, Kanagawa 236-0004 Japan; 10 Yokohama City University Center for Novel and Exploratory Clinical trials; 1-1-1, Fukuura, Kanazawa-ku, Yokohama, Kanagawa 236-0004 Japan; 11 Gastroenterology Division, Hadano Red Cross Hospital; 1-1-1, Tatenodai, Hadano, Kanagawa 257-0017 Japan

**Keywords:** Hepatocellular carcinoma, Hepatic arterial infusion chemotherapy, Cisplatin, Sorafenib, Sequential treatment

## Abstract

**Background:**

The efficacy of hepatic arterial infusion chemotherapy (HAIC) for advanced hepatocellular carcinoma (HCC) remains unclear. We conducted a multi-center randomized phase II study comparing a sequential HAIC-sorafenib regimen versus sorafenib alone as an initial therapy for HCC.

**Methods:**

Patients were randomly assigned (ratio, 1:1) to receive sequential HAIC with cisplatin followed by sorafenib (HAIC group, *n* = 35) or sorafenib alone (sorafenib group, *n* = 33) as an initial therapy. The primary endpoint was the one-year survival rate. Secondary endpoint included overall survival (OS), the 2-year survival rate, the time-to-progression (TTP), the objective response rate (ORR), the disease control rate (DCR), and safety.

**Results:**

For the primary endpoint, the one-year survival rates were 46% in the HAIC group and 58% in the sorafenib group. The median OS period was 10.0 months (95% CI, 7.0–18.8) in the HAIC group and 15.2 months (95% CI, 8.2–19.7) in the sorafenib group (hazard ratio [HR], 1.08; 95% CI, 0.63 to 1.86, *P* = 0.78). The median TTP, ORR and DCR in the HAIC group were 2.8 months (95% CI, 1.7–5.5), 14.3, and 45.7%, respectively, while those in the sorafenib group were 3.9 months (95% CI, 2.3–6.8), 9.1, and 45.5%, respectively. No unexpected adverse events related to HAIC or sorafenib were observed in either group.

**Conclusions:**

Sequential HAIC with cisplatin and sorafenib does not improve the survival benefit, compared with sorafenib alone, when used as an initial therapy for advanced HCC. However, this study was underpowered in regard to its primary and secondary endpoints, so the results should be interpreted with caution.

**Trial registration:**

UMIN ID 000006147, registration data: August 11, 2011.

## Background

Hepatocellular carcinoma (HCC) is one of major causes of death from cancer in the world [1]. On the basis of classification according to the Barcelona Clinic Liver Cancer (BCLC) staging system, patients with intermediate-stage disease showing disease progression after transarterial chemoembolization (TACE) and those with advanced-stage disease have an exceedingly poor prognosis [2, 3]. Sorafenib, an oral multikinase inhibitor, has been demonstrated to improve the overall survival (OS) in advanced HCC patients in two placebo-controlled, randomized phase III studies [4, 5]. Although sorafenib has been recommended as a first-line treatment not only for advanced-stage HCC patients, but also for intermediate-stage patients who are not expected to benefit from TACE [6], the discontinuation rates because of drug-related adverse events (AEs) were over 40% in two prospective postmarketing surveillance studies performed in Japan [7, 8]. Furthermore, the tolerability of sorafenib among elderly HCC patients also seems to be relatively low [9].

Hepatic arterial infusion chemotherapy (HAIC), in which high local concentrations of antitumor agents are achieved with reduced systemic side effects, has been used as a palliative treatment modality. HAIC has been used to treat patients with advanced HCC in Japan and other Asian countries as it has been shown to yield good disease control rates and is tolerated well [10–14]. The CDDP plus 5-fluorouracil (5-FU) [10, 11] and 5-FU plus interferon [12, 13] regimens are commonly used combination regimens for patients with HCC; however, use of these regimens involves the complex management of the implanted catheter system. However, no prospective or randomized studies have been conducted, and its efficacy remains unclear. HAIC with cisplatin alone, which accounts for 11% of all HAIC-treated cases of HCC [15] in Japan, is commonly used for treating patients with advanced HCC. Cisplatin is an anticancer agent that acts in a concentration- and time-dependent manner, and it was shown by Court et al. [16] that 48.4% (range: 34.2–55%) of the administered dose is taken up by liver tumors by first-pass kinetics following intravenous injection of ^195m^cisplatin. Therefore, following selective single-dose administration of cisplatin via the hepatic artery, it is expected that cisplatin would accumulate at even higher concentrations in liver tumors, to exert high therapeutic efficacy. One of the representative HAIC regimens in Japan uses a fine powder formulation of cisplatin (IA call®; Nippon Kayaku, Tokyo, Japan) adjusted to an approximately three-fold concentration of cisplatin (1.4 mg/mL) in an aqueous solution, compared with conventional cisplatin formulations (0.5 mg/mL). The efficacy and tolerability of this formulation were found to be acceptable in patients with unresectable HCC [14].

There are no randomized trials comparing HAIC and sorafenib as a first-line therapy for advanced HCC; therefore, the positioning of HAIC in the treatment of advanced HCC continues to be debated. In this multi-center randomized phase II study, we compared the efficacy and safety of a sequential HAIC treatment using cisplatin followed by sorafenib versus sorafenib alone—a global standard option—as an initial therapy for advanced HCC.

## Methods

### Study design and patients

This randomized, open-label phase II trial was performed at 8 institutes in Japan and was approved by each institute’s review board. Written informed consent was obtained from all the patients. This study was performed according to the guidelines of the Helsinki Declaration and the CONSORT guidelines, and was registered with the University Hospital Medical Network Clinical Trial Registry (UMIN ID 000006147).

Eligible patients were diagnosed as having HCC based on the findings of histopathological examinations, imaging examinations, and/or clinical diagnosis in accordance with the American Association for the Study of Liver Diseases criteria. The eligibility criteria included patients who were unlikely to benefit from surgical resection or locoregional treatment, and were aged 20 years or over, and had a life expectancy of 12 weeks or more, Eastern Cooperative Oncology Group (ECOG) performance status score of 0 or 1, and a Child-Pugh score of 7 or less. Previous treatment was terminated at least 4 weeks before this study entry. Patients were required to have adequate renal, hematological, and hepatic function, as indicated by a neutrophil count of 10^3^/μL or greater, a platelet count of 50 × 10^3^/μL or greater, a hemoglobin concentration of 8.5 g/dL or more, a total bilirubin concentration of 3.0 mg/dL or less, a serum creatinine concentration of 1.5 mg/dL or less, an aspartate and alanine aminotransferase concentration of five-times the upper limit of normal or less, or a serum amylase concentration of two-times the upper limit of normal or less. Patients with extrahepatic metastasis, in whom the lesions were determined by the attending physicians as not being determinants of the prognosis, were allowed to participate in this study. The exclusion criteria were as follows: previous treatment using sorafenib or cisplatin, the presence of serious cardiovascular disease, the presence of esophageal gastric varices and/or gastroduodenal ulcers requiring treatment, the presence of other advanced carcinomas, pregnancy, and extrahepatic lesions which will affect their prognosis.

Patients were randomly assigned in a 1:1 ratio to receive sequential HAIC with cisplatin followed by sorafenib upon progression (HAIC group) or sorafenib alone (sorafenib group) as an initial therapy, using minimization method with a random element using (1) institute, (2) presence of portal vein tumor embolism, (3) presence of extrahepatic lesion. Random number was generated by SAS 9.3 (SAS Institute, Cary, NC, USA). Eligible patients were stratified by the presence or absence of portal vein thrombosis or extrahepatic spreading and the institution at which they received treatment. They were enrolled by their doctor in charge and randomization is performed centrally by Data Center, Yokohama City University Medical Center, using Web registration system.

### Procedures

All treatments administered during the trial were open labelled. In the HAIC group, the catheter was inserted into the femoral artery using the Seldinger technique [17] and advanced into the celiac artery; then a microcatheter was advanced into the proper hepatic artery for the chemoinfusion. A fine powder formulation of cisplatin (cisplatin powder) was dissolved in saline solution that had been heated to 50 °C. This solution was then administered at a dose of 65 mg/m^2^ over a period of about 30 min [18]. This treatment was repeated at an interval of 4 to 6 weeks until intolerable toxicity or radiological progressive disease (PD). HAIC treatment also halted according to a planned switch criterion—Clinical PD—based on the alpha-fetoprotein (AFP) and des-gamma carboxyprothrombin (DCP) levels. Clinical PD was defined as follows: an increase of 20% or more in both the AFP and DCP levels compared with the levels at the time of the previous treatment, an increase in the AFP level of 20% or more of an AFP level equivalent to more than 500 ng/mL at the time of the previous treatment, or an increase in the DCP level of 20% or more of a DCP level equivalent to more than 500 mAU/mL at the time of the previous treatment. These definitions were derived from the AFP and DCP levels of PD patients who achieved SD after the first session of HAIC but exhibited PD after the second session (personal communication). Patients who meet these withdrawal criteria received sorafenib treatment within 14 days after the determination PD. Patients whose HAIC treatments were terminated because of adverse events received sorafenib treatment once their adverse effects had recovered to a grade of less than 1.

In the sorafenib group, sorafenib was initiated at a dose of 400 mg twice daily. An initial dosage reduction to 400 mg once daily was allowed in some elderly patients or some patients decreasing hepatic reserves. If intolerable toxicities appeared, treatment interruptions and dose reduction were also allowed.

This study protocol was terminated if radiological PD was confirmed after sorafenib treatment in both groups. An attending physician could decide the post-protocol treatment as to whether sorafenib treatment continue, or switch to / use together with other treatment options.

### End points

The primary end point of the study was the one-year survival rate, and the secondary end point were overall survival (OS), the 2-year survival rate, the time to progression (TTP), the objective response rate (ORR), the disease control rate (DCR), and safety. OS was measured from the date of randomization until death or the last consultation day. TTP was measured from the date of the first HAIC session or starting sorafenib treatment until the date of confirmation of the tumor progression radiologically or deterioration of the obvious patients’ condition. The tumor response was evaluated on the basis of the modified Response Evaluation Criteria for Solid Tumors (mRECIST) [19], based on dynamic computed tomography (CT) or magnetic resonance imaging (MRI) performed every 4–6 weeks during sorafenib treatment and at the end of 4 weeks after each HAIC session. The toxicities were evaluated based on the Common Terminology Criteria for Adverse Events (CTCAE 4.0).

### Statistical analysis

Efficacy was analyzed in the intent-to-treat population, defined as all randomly assigned patients, and safety was analyzed for all the patients who received each treatment. The sample size calculations were based on the following: with a null one-year survival rate of 43.5%, and an expected one-year survival rate of 57.4% in patients receiving HAIC with cisplatin followed by sorafenib therapy, to obtain a clinically meaningful improvement in the median OS from 11 months in the sorafenib group to 15 months in the HAIC group with an 80% power to detect a superior survival outcome in the HAIC group using a one-sided alpha level of 5%, it was determined that 42 patients should be assigned to each group in a random manner. Statistical analyses were performed using SAS 9.3 (SAS Institute, Cary, NC, USA). Continuous parameters were expressed as the mean ± standard deviation or the medians and ranges, and categorical variables were expressed as the numbers and percentages or the frequencies. The assessment of differences in the baseline features of patients between both groups was determined using the Student *t*-test for continuous variables and the χ^2^ test for categorical variables. The statistical significance of differences in the ORR and DCR between the two groups were assessed using the χ^2^ test for categorical variables. The hazard ratio (HR) and 95% confidence interval (CI) were determined to estimate the efficacies of the sorafenib and HAIC treatments, respectively. A two-side *P* value of less than 0.05 was considered statistically significant. Survival curves for OS and TTP were plotted using the Kaplan-Meier method, and any significant differences between the two groups were compared using the log-rank test. Variables that reached a *P* values of less than 0.05 were regarded as significant in the univariate cox regression analysis.

## Results

### Patients and treatment administration

From August 2011 through November 2014, 70 patients were enrolled in this study from 8 institutions. Because of slow accrual, this study closed prematurely with a reduced sample size, unlike the planned enrolled number of patients. All the patients were randomly allocated to the HAIC group (36 patients) or the sorafenib group (34 patients). One patient was excluded from each group after they withdrew consent. Follow-up was continued until October 2015, corresponding to 1 year after the enrollment of the last patient. Table 1 shows the baseline characteristics of the patients at the start of this study; the characteristics were evenly balanced between the treatment groups.

**Table 1:** Patients’ characteristics in the sorafenib group and the HAIC group

Variables	Sorafenib group (*n* = 33)	HAIC group (*n* = 35)	*P*
Sex			
Male/Female	27 / 6	28 / 7	1.000
Mean age (years)	70.9 ± 9.1	72.0 ± 7.0	0.571
Cause			
HCV / HBV / NBNC	20 / 4 / 10	21 / 3 / 11	0.308
Previous treatment			
Ablation/TACE+TAE/None	1/20/12	2/24/9	0.308
BCLC			
A / B / C	2 / 13 /18	2 /14 /19	0.994
UICC			
II/IIIA/IIIB/IIIC/IVA/IVB	14/3/7/1/3/5	15/2/6/2/4/6	0.802
Child-Pugh class			
5 / 6 / 7	10 /19 /4	20 / 11 / 4	0.061
PVTT			
yes / no	22 / 11	21 / 14	0.621
Extrahepatic metastasis			
yes / no	8 / 25	10 / 25	0.786
Gastroesophageal varices			
yes / no	10 / 23	5 / 30	0.099
AFP (ng/mL) ^a^	216.7 (5–161,160)	67.3 (2–281,600)	0.688
DCP (mAU/mL) ^a^	1068 (14–272,000)	335 (12–99,800)	0.364

^a^Data are the median values

*HCV* hepatitis C virus, *HBV* hepatitis B virus, *BCLC* Barcelona Clinic Liver Cancer, *UICC* union for international cancer control, *PVTT* portal vein tumor thrombosis, *AFP* alpha-fetoprotein, *DCP* des-gamma carboxy-prothrombin

In the HAIC group, the median number of HAIC treatments was 2 sessions (range, 1–11 sessions). Tumor-stage improvement was achieved in 4 patients, and they received subsequent conversion options (resection in one patient, RFA in one, and TACE in two patients). Eight patients did not receive subsequent sorafenib treatment because of a deterioration in their general conditions (*n* = 3), a decrease in their hepatic functional reserves (*n* = 2), drug-related toxicities (*n* = 1), a change in treatment to ablation after consultation with the patient (*n* = 1), and the patient’s request (*n* = 1). Twenty-three (66%) of the 35 patients in the HAIC group received subsequent sorafenib treatment upon radiological PD (*n* = 16) or clinical PD (*n* = 7). The median duration between the start of HAIC treatment and subsequent sorafenib treatment was 2.6 months. Eight-patients initially received 400 mg of sorafenib twice daily, while 15 patients received less than 800 mg daily. The median duration of sorafenib treatment was 2.8 months (range, 0.2–16.5 months), and the median average daily dose (range) was 400 mg (325–800 mg). The study protocol was terminated upon radiological PD (*n* = 16) or drug-related toxicities (*n* = 6); however, one patient terminated sorafenib treatment upon the achievement of a CR.

In the sorafenib group, the median duration of sorafenib treatment was 2.7 months (range, 0.1–27.3 months). Of the 33 patients, 15 patients initially received 400 mg twice daily, while 18 patients received less than 800 mg daily. The median average daily dose (range) was 400 mg (227–800 mg). The study protocol was terminated upon radiological PD (*n* = 22) or drug-related toxicities (*n* = 11).

### Efficacy

**Fig. 1:**
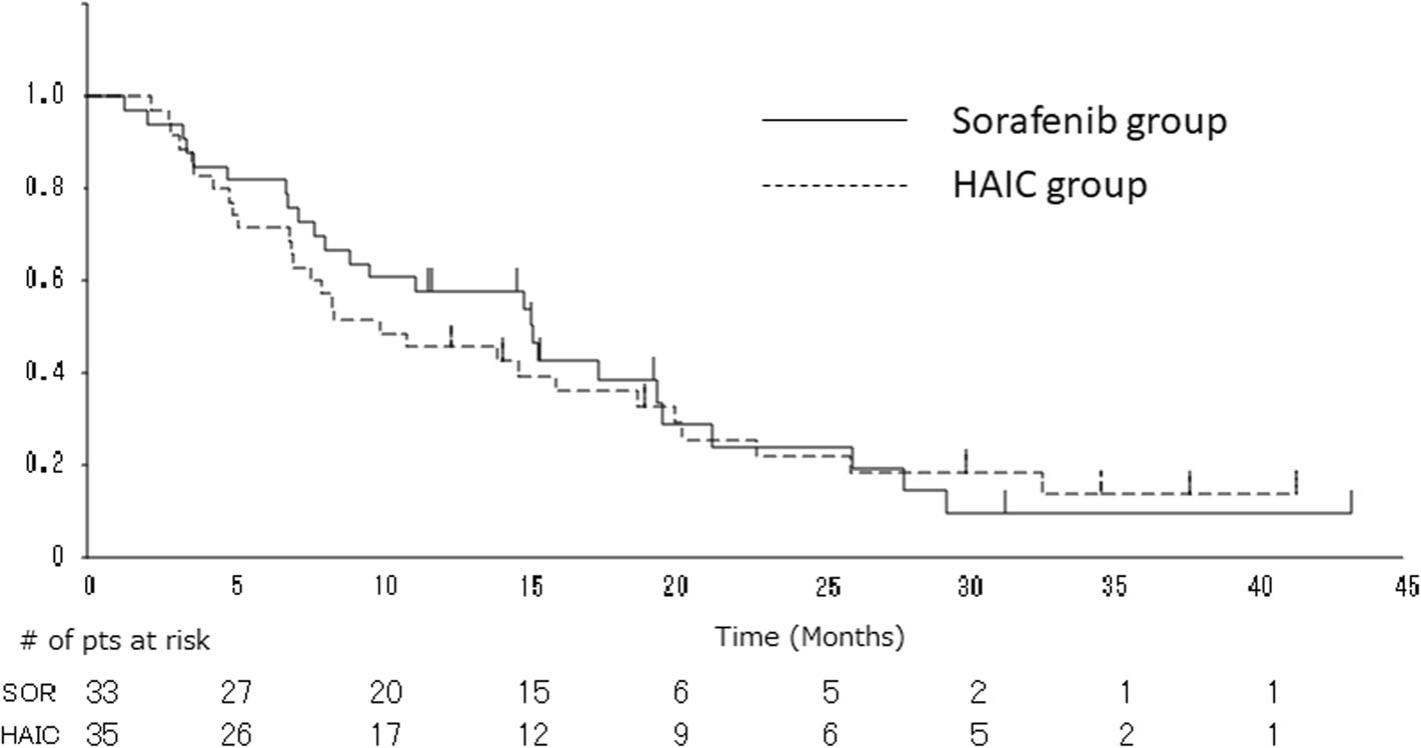
Comparison of overall survival between the sorafenib group (solid line) and the HAIC group (dotted line). HAIC, hepatic arterial infusion chemotherapy

**Fig. 2:**
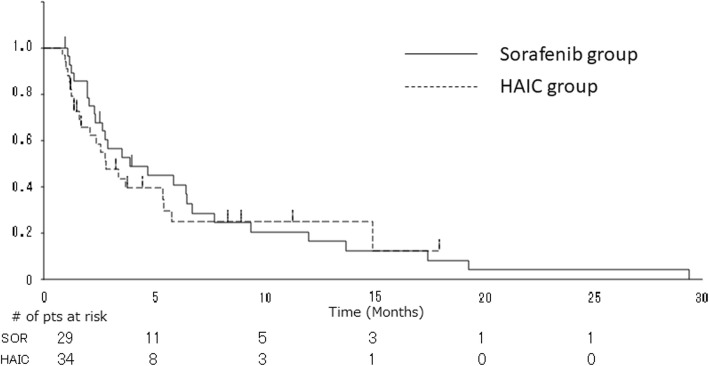
Comparison of time to progression between the sorafenib group (solid line) and the HAIC group (dotted line). HAIC, hepatic arterial infusion chemotherapy

The one-year survival rate, which was the primary end point of the study, was 46% in the HAIC group, which was not superior to the rate of 58% in the sorafenib group. The 2-year survival rates were similar: 22% in the HAIC group, and 24% in the sorafenib group. The ORR tended to be higher in the HAIC group than in the sorafenib group (14.3% versus 9.1%, respectively; *P* = 0.710; Table 2); however, the DCR was similar: 45.7% in the HAIC group, and 45.5% in the sorafenib group. The median OS in the HAIC group was 10.0 months (95% CI, 7.0–18.8), which was not significantly different from that in the sorafenib group (median, 15.2 months; 95% CI, 8.2–19.7 months) (HR, 1.08; 95% CI, 0.63–1.86; *P* = 0.78; Fig. 1). The median TTP for the HAIC treatment was 2.8 months (95% CI, 1.7–5.5 months), while that for the sorafenib treatment in the sorafenib group was 3.9 months (95% CI, 2.3–6.8 months) (HR, 1.17; 95% CI, 0.65–2.10; *P* = 0.60; Fig. 2). In the HAIC group, 23 patients received subsequent sorafenib treatment after HAIC failure; the ORR and DCR of the group that received subsequent sorafenib treatment were 4.3 and 34.8%, respectively (Table 2), and the median TTP of the group that received subsequent sorafenib treatment was 4.2 months (95% CI, 2.6–6.5 months).

**Table 2:** Effectivity due to sorafenib treatment in the sorafenib group and the HAIC group

mRECIST	Sorafenib group (%) (*n* = 33)	HAIC group (%)	
		HAIC	sorafenib
		(*n* = 35)	(*n* = 23)
CR	0 (0)	0 (0)	1 (4)
PR	3 (9)	5 (14)	0 (0)
SD	12 (36)	11 (31)	7 (30)
PD	15 (46)	16 (46)	14 (61)
NE	3 (9)	3 (9)	1 (4)
ORR	3 (9)	5 (14)	1 (4)
DCR	15 (46)	16 (46)	8 (35)

*HAIC* hepatic arterial infusion chemotherapy, *CR* complete response, *PR* partial response, *SD* stable disease, *PD* progressive disease, *NE* not evaluable, *ORR* objective response rate, *DCR* disease control rate

**Fig. 3:**
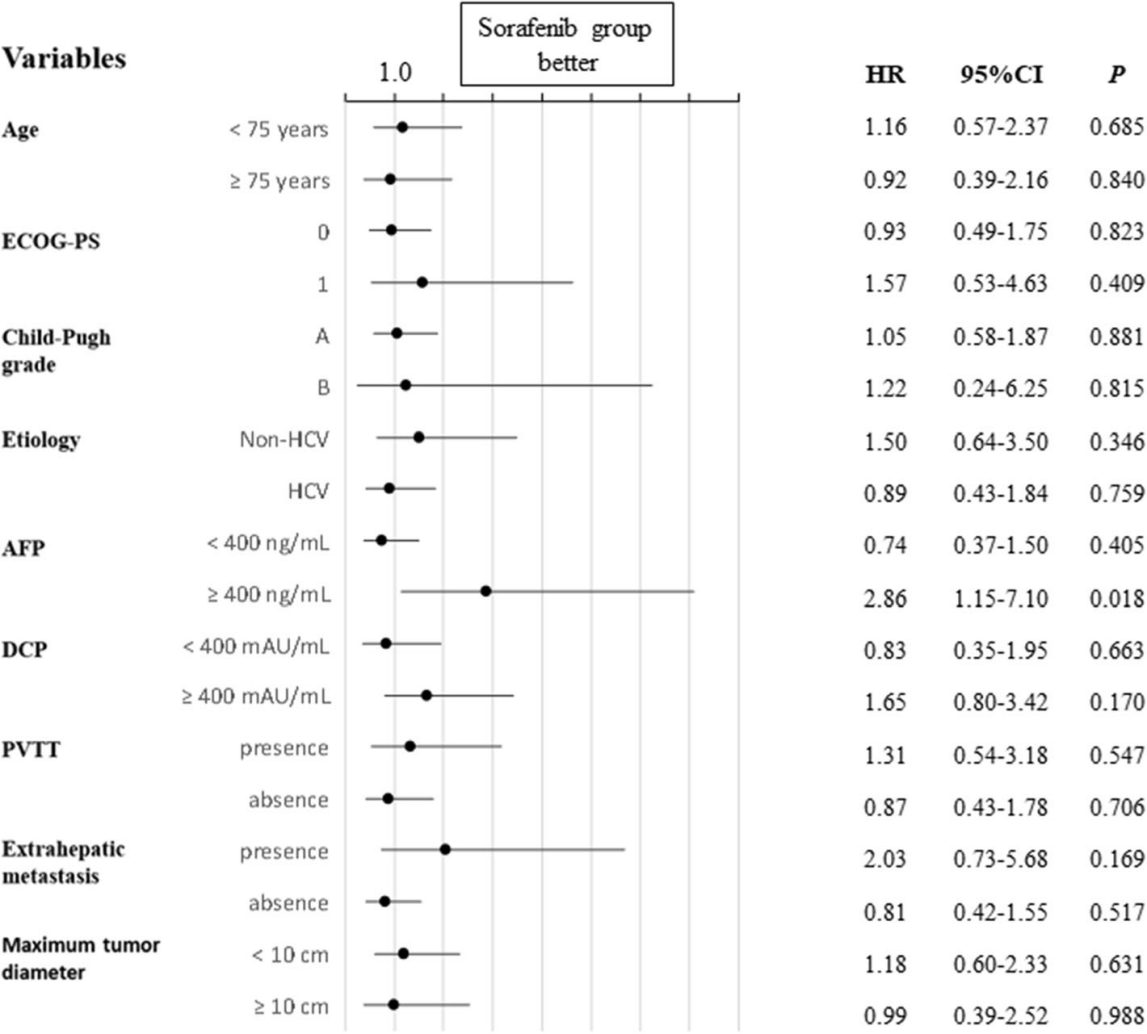
Forest plots showing a subgroup analysis of overall survival. ECOG-PS, Eastern Cooperative Oncology Group-Performance Status; HCV, hepatitis C virus; AFP, alpha-fetoprotein; DCP, des-gamma carboxy-prothrombin; PVTT, portal vein tumor thrombosis. The HRs were calculated by univariate cox regression analysis

Subgroup analyses of OS according to the allocation factors, including disease etiology (hepatitis C or non-hepatitis C), portal vein tumor thrombosis (presence or absence), and extrahepatic metastasis (presence or absence), did not show any superiorities of HAIC to sorafenib between the two groups (Fig. 3); however, in patients with a baseline AFP ≥400 ng/mL, the OS of the sorafenib group was significantly better than that of the HAIC group (HR, 2.86; 95% CI, 1.15–7.10; *P* = 0.018).

Among the 18 patients with extrahepatic metastasis, 10 were included in the HAIC group and the remaining 8 in the sorafenib group. Of the 10 patients in the HAIC group, the treatment response was rated as PR and SD in 1 and 3 patients, respectively, whereas PD was noted in 5 patients and the treatment effect was not evaluated in 1 patient. On the other hand, of the 8 patients in the sorafenib group, the response was rated as SD in 4 patients, PD was noted in 3 patients, and the effect was not evaluated in 1 patient. The median TTP was 62 days in the HAIC group and 82 days in the sorafenib group, with no significant difference between the two groups (*p* = 0.945).

### *Safety* (Table 3)

In the HAIC group, the most frequently observed adverse events were an elevated aspartate or alanine aminotransferase level (9%). One patient (3%) discontinued HAIC because of unacceptable drug-related toxicities (total bilirubin elevation). In 23 patients who received subsequent sorafenib treatment, six patients (26%) discontinued sorafenib treatment because of unacceptable drug-related toxicities. The frequently observed adverse events of grade 3 or higher were hypertension (35%), an elevated aspartate or alanine aminotransferase level (26%), hand-foot skin reaction (26%), and an elevated lipase level (17%).

**Table 3:** Serious adverse events due to protocol treatment in the sorafenib group and the HAIC group

	Sorafenib group (*n* = 33)	HAIC group (*n* = 35)	
		HAIC	sorafenib
			(*n* = 23)
Adverse events	Grade 3/4	Grade 3/4	
	*n* (%)	*n* (%)	
Non-hematological			
Mucositis oral	1 (3)		1 (4)
Hoarseness	0 (0)		1 (4)
Hand-foot skin reaction	5 (15)		6 (26)
Rash	2 (6)		0 (0)
Anorexia	1 (3)		2 (9)
Diarrhea	1 (3)		0 (0)
Bleeding	1 (3)		1 (4)
Fatigue	1 (3)		1 (4)
Hypertension	6 (18)		8 (35)
Somnolence	1 (3)		0 (0)
Weight loss	1 (3)		0 (0)
Abdominal distension	0 (0)		1 (4)
Encephalopathy	0 (0)	1 (3)	0 (0)
Nausea	0 (0)	1 (3)	0 (0)
Hiccups	0 (0)	1 (3)	0 (0)
Absence seizure	0 (0)	1 (3)	0 (0)
Hydrocele testis	0 (0)	1 (3)	0 (0)
Hematological			
Thrombocytopenia	3 (9)		0 (0)
Transaminase elevation	7 (21)	3 (9)	6 (26)
Total bilirubin elevation	2 (6)	1 (3)	2 (9)
Creatinine elevation	0 (0)		0 (0)
Amylase elevation	1 (3)		0 (0)
Lipase elevation	9 (27)		4 (17)
Liver failure	1 (3)		0 (0)
Hypoalbuminemia	0 (0)		1 (4)
Hyponatremia	1 (3)		0 (0)
Hypophosphatemia	2 (6)		0 (0)
Others			
Portal thrombosis	0 (0)	1 (3)	0 (0)

*HAIC* hepatic arterial infusion chemotherapy

In the sorafenib group, eleven patients (33%) discontinued sorafenib treatment because of unacceptable drug-related toxicities. The frequently observed adverse events of grade 3 or higher were an elevated lipase level (27%), an elevated aspartate or alanine aminotransferase level (21%), hypertension (18%), and hand-foot skin reaction (15%).

### Post-protocol treatment

Post-protocol treatment was given to 18 patients (51%) in the HAIC group and 29 patients (88%) in the sorafenib group (Table 4). Among the patients receiving post-protocol options, the main option (≥10 patients) was sorafenib continuation (29%) in the HAIC group, while HAIC (55%) and sorafenib continuation (33%) were administered in the sorafenib group.

**Table 4:** Post protocol treatment in the sorafenib group and the HAIC group

Post protocol treatment	Sorafenib group (%)	HAIC group (%)
Absent	4 (12)	17 (49)
Present	29 (88)	18 (51)
sorafenib continuation	11 (33)	10 (29)
Resection	0 (0)	1^a^ (3)
RFA	2 (6)	1^b^ (3)
TACE	5 (15)	5^c^ (14)
HAIC	18 (55)	3 (9)
Others	1^d^ (3)	2^e^ (6)

*HAIC* hepatic arterial infusion chemotherapy, *RFA* radiofrequency ablation, *TACE* transarterial chemoembolization; Resection as a conversion option were administered in one patient ^a^, RFA in one ^b^, and TACE in two of five patients ^c^; ^d^, One patient participated in a clinical trial examining tivantinib; ^e^, One patient participated in a clinical trial examining tivantinib and another one received percutaneous ethanol injection treatment (PEIT)

## Discussion

Our study examined whether sequential HAIC with cisplatin powder and sorafenib improve the survival benefit compared with sorafenib alone as an initial therapy for advanced HCC. Since the one-year survival rate, a primary end point of the study, tended to be lower in the HAIC group than in the sorafenib group, our results suggest that sorafenib should be used as first-line therapy for advanced HCC. To the best of our knowledge, this is the first randomized study comparing an HAIC regimen versus sorafenib as an initial therapy for advanced HCC.

HAIC has been frequently administered to patients with advanced HCC in Asian countries, especially those with PVTT [10–13]. Although its survival benefit remains unclear because of a lack of randomized control trials, a favorable disease control rate and tolerability were reported with variable regimens, including cisplatin plus low-dose 5-FU [10, 11], 5-FU plus interferon [12, 13], and monotherapy with cisplatin powder [14]. The outcome in a nationwide survey in Japan seems favorable [15], with a reported response rate of 45.9% and a disease control rate of 76.5%. Advanced HCC patients who respond to HAIC have a prolonged OS, with a reported median overall survival (MST) of 40.7 months in responders to low-dose 5-FU and cisplatin [11]. We previously reported the efficacy of HAIC with cisplatin powder for advanced HCC with PVTT, and the MST of the responders was 37.3 months [18]. Thus, we planned to encourage the positioning of HAIC in the treatment of advanced HCC and conducted a multi-center randomized phase II study comparing sequential HAIC treatment using cisplatin powder followed by sorafenib versus sorafenib alone—a global standard option—as an initial therapy for advanced HCC.

Sorafenib, acting on the inhibition of a multikinase involved in tumor cell signaling, proliferation, angiogenesis, and apoptosis, is the only proven, global standard treatment for advanced HCC patients [20]. Sorafenib treatment in two prospective post-marketing analyses for Japanese patients [7, 8] was well tolerated; however, the discontinuation rate because of drug-related adverse events (AEs) was over 40% in both studies despite a reduction in the median daily dose of sorafenib from 614 mg to 419 mg. A high discontinuation rate because of intolerance, particularly in elderly patients, has been reported [9, 21]. In the present study, the ORR tended to be lower in the sorafenib group than in the HAIC group; however, the median TTP and OS tended to be higher in the sorafenib group than in the HAIC group. Subgroup analyses of OS according to the pretreatment characteristics did not show any superiorities of HAIC to sorafenib, but sorafenib showed a favorable HR compared with HAIC in the subset of patients with a baseline AFP ≥400 ng/mL, suggesting that sorafenib should be used as a first-line therapy for advanced HCC.

Patients with extrahepatic metastasis, irrespective of the prognosis, were eligible for inclusion in this study. In our clinical practice, the prognosis in patients with uncontrolled intrahepatic lesions seemed not to be affected by small extrahepatic metastases in the lymph nodes, lungs, or adrenal glands. Some studies have reported that effective treatments for intrahepatic lesions are of benefit to HCC patients with extrahepatic metastasis [22, 23]. Although it is difficult to arrive at any conclusion regarding the efficacy of the treatment in patients with extrahepatic metastasis from this study because of the small sample size, there was no significant difference in the TTP between the two groups (*p* = 0.945).

Some reasons why sequential HAIC with cisplatin powder and sorafenib did not improve the survival benefit compared with sorafenib alone as an initial therapy for advanced HCC may be as follows: 1) the effectiveness of post-protocol treatments after sorafenib failure may have prolonged the post-progression survival (PPS), especially in the sorafenib group; and 2) the relatively high rate of protocol treatment discontinuation after HAIC-failure may have resulted in a loss of appreciation of the antitumor effect of the subsequent sorafenib treatment in the HAIC group. Regarding the former possibility, 88% of the patients in the sorafenib group received post-protocol treatment options, including HAIC with cisplatin powder which was administered in 55% of the patients of the sorafenib group. Therefore, crossover of the treatment options, including HAIC and sorafenib, could minimize the difference in the OS between the two groups. Since HAIC using low-dose 5-FU and cisplatin has been shown to exhibit moderate antitumor efficacy for advanced HCC patients after sorafenib failure [24], these post-protocol treatment options may prolong the PPS after sorafenib failure, thereby contributing to a longer OS in the sorafenib group [25, 26]. Regarding the latter possibility, 8 (23%) of the 35 patients did not start subsequent sorafenib treatment after HAIC-failure because of a deterioration in their general conditions, a decrease in hepatic functional reserve, and drug-related toxicities, potentially losing any advantage of sorafenib treatment in the HAIC group.

Because HCC has heterogeneous intratumor characteristics [27, 28], multidisciplinary treatments, including combination or sequential treatments, might have some impact on the treatment of advanced HCC patients. In our sequential setting, HAIC with cisplatin powder followed by sorafenib did not improve the survival benefit compared with sorafenib alone as an initial therapy for advanced HCC. Regarding combined settings, two prospective randomized controlled trials of sorafenib plus HAIC using low-dose 5-FU and cisplatin (low-dose FP therapy) versus sorafenib alone (SILIUS study: NCT01214343) [29] and of sorafenib plus HAIC using cisplatin powder versus sorafenib alone (CDDP-Sor- randomized Phase II [rP2] study: UMIN000005703) [30] for patients with advanced HCC have been recently reported. In the SILIUS study, the addition of HAIC to sorafenib alone did not have any effect on the OS in this Phase III trial [29], and a superior OS was limited to the subgroup of patients with main portal vein invasion. In the CDDP-Sor-rP2 study, a favorable effect on OS has been reported for the addition of HAIC using cisplatin powder to sorafenib [30]; the stratified HR (95% CI) was 0.60 (0.38–0.96) (*P* = 0.031), and a Phase III trial is anticipated.

The present study had some limitations; it was mainly attributable to the difficulty in recruiting suitable patients, and the number of enrolled patients was less than that required by the study design. Our HCC patients could select any of the following treatment regimens: 1) sorafenib treatment, 2) HAIC with other chemotherapeutic agents, or 3) participation in other clinical trials involving the use of other multikinase inhibitors or anticancer agents. With regard to HAIC with other regimens, we do not use any of the other commonly used agents for HAIC in Japan, such as low-dose FP therapy or 5-FU plus interferon-α (FAIT therapy). A phase II study comparing the efficacy of FAIT therapy to those of low-dose FP therapy or intraarterial cisplatin as a reference group did not show any efficacious differences between the treatment groups [31], suggesting that intraarterial cisplatin powder may be comparable to low-dose FP therapy or FAIT therapy. Another limitation was that the HAIC could be halted in the event of clinical PD, based on our personal communications. In the present study, the second session of HAIC could be avoided in 7 patients and the treatment was switched to sorafenib after the first session of HAIC. The median OS of these 7 patients was 10.9 months (2.8–15.8 months), which was comparable to the median OS (10 months) of all patients in the HAIC group, suggesting that our definition of “clinical PD” is unlikely to be disadvantageous for our patients.

## Conclusions

A survival benefit of HAIC with cisplatin powder followed by sorafenib was not demonstrated. Further studies on determining an appropriate treatment regimen in HAIC may be important, including a combination setting with sorafenib or a sequential setting after sorafenib failure.

## Data Availability

The datasets generated and/or analysed during the current study are not publicly available because it was not a big project study to have such a public datasets, but are available from the corresponding author on reasonable request.

## Abbreviations

AEs: Adverse events
AFP: Alpha-fetoprotein
BCLC: Barcelona Clinic Liver Cancer
CI: Confidence interval
CR: Complete response
CT: Computed tomography
CTCAE: Common Terminology Criteria for Adverse Events
DCP: Des-gamma carboxyprothrombin
DCR: Disease control rate
ECOG: Eastern Cooperative Oncology Group
HAIC: Hepatic arterial infusion chemotherapy
HCC: Hepatocellular carcinoma
HR: Hazard ratio
mRECIST: modified Response Evaluation Criteria for Solid Tumors
MRI: Magnetic resonance imaging
MST: Median overall survival
ORR: Objective response rate
OS: Overall survival
PD: Progressive disease
PPS: Post-progression survival
PVTT: Portal vein tumor thrombosis
RFA: Radiofrequency ablation
TACE: Transarterial chemoembolization
TTP: Time to progression
